# Geometric and Inertial Properties of the Pig Head and Brain in an Anatomical Coordinate System

**DOI:** 10.1007/s10439-023-03294-y

**Published:** 2023-06-26

**Authors:** Nikoo Soltan, Gunter P. Siegmund, Peter A. Cripton, Claire F. Jones

**Affiliations:** 1 Department of Mechanical Engineering, The University of British Columbia, Vancouver, BC Canada; 2 Orthopaedic and Injury Biomechanics Group, ICORD, Vancouver, BC Canada; 3 MEA Forensic Engineers & Scientists, Laguna Hills, CA USA; 4 School of Kinesiology, The University of British Columbia, Vancouver, BC Canada; 5 School of Biomedical Engineering, The University of British Columbia, Vancouver, BC Canada; 6 School of Electrical and Mechanical Engineering, The University of Adelaide, Adelaide, SA Australia; 7 Adelaide Spinal Research Group, Centre for Orthopaedic & Trauma Research, The University of Adelaide, Adelaide, SA Australia; 8 Department of Orthopaedics & Trauma, Royal Adelaide Hospital, Adelaide, SA Australia

**Keywords:** Porcine model, Center of mass, Moment of inertia, Atlanto-occipital joint, Mass, Volume, Preclinical injury model

## Abstract

Porcine models in injury biomechanics research often involve measuring head or brain kinematics. Translation of data from porcine models to other biomechanical models requires geometric and inertial properties of the pig head and brain, and a translationally relevant anatomical coordinate system (ACS). In this study, the head and brain mass, center of mass (CoM), and mass moments of inertia (MoI) were characterized, and an ACS was proposed for the pre-adolescent domestic pig. Density-calibrated computed tomography scans were obtained for the heads of eleven Large White × Landrace pigs (18–48 kg) and were segmented. An ACS with a porcine-equivalent Frankfort plane was defined using externally palpable landmarks (right/left frontal process of the zygomatic bone and zygomatic process of the frontal bone). The head and brain constituted 7.80 ± 0.79% and 0.33 ± 0.08% of the body mass, respectively. The head and brain CoMs were primarily ventral and caudal to the ACS origin, respectively. The mean head and brain principal MoI (in the ACS with origin at respective CoM) ranged from 61.7 to 109.7 kg cm^2^, and 0.2 to 0.6 kg cm^2^, respectively. These data may aid the comparison of head and brain kinematics/kinetics data and the translation between porcine and human injury models.

**Supplementary Information:**

The online version contains supplementary material available at 10.1007/s10439-023-03294-y.

## Introduction

Large animal models are a valuable translational resource in biomechanics research [10, 17, 30]. Among these models, pigs are used in several injury applications including spinal cord injury [13, 19, 30], traumatic brain injury (TBI) [8, 32], whiplash injury [26, 36], and other musculoskeletal disorders of the spine [22, 25, 27]. Porcine models of head, brain and/or neck injury often involve applying a scaled mechanical perturbation [8, 10, 14] and measuring three-dimensional (3D) head kinematics using instrumentation mounted to the head or head-coupled test apparatus [8, 32, 36]. However, the pig head/brain geometric and inertial properties are not adequately characterized across the range of animal sizes used in these studies, which could limit the accurate scaling of loading conditions. Additionally, no standardized location for reporting head kinematics has been established for the pig, which limits comparisons between porcine and other models.

Head kinematics in other human biomechanical surrogates and human subjects are often reported at the center of mass (CoM) or the atlanto-occipital joint (AOJ) [33, 39]. Therefore, translation of kinematics data from porcine studies can be aided by defining the location of the pig head and brain CoMs, and the AOJ. Additionally, since injury severity can be dependent on the impact direction relative to anatomical features (e.g., orientation of spine axis to the brain) [8, 31], developing an anatomically-relevant head coordinate system for the quadrupedal pig can be used to translate kinematics data to bipedal human models. If defined by palpable bony landmarks, such an anatomical coordinate system (ACS) could be particularly valuable in injury biomechanics studies as it eliminates the need for 3D medical imaging and analysis, and allows real-time location of the head in the laboratory coordinate system. Furthermore, characterizing the mass moments of inertia (MoI) of the pig head can enable calculation of forces and moments at the AOJ and aid the translational application of kinetics data from porcine models.

The brain mass of various domestic strains have previously been reported for neonate, adolescent, and adult (female and male) pigs [20, 24]. However, pre-adolescent (8–13 weeks [6, 7]) is the most common developmental stage used for domestic porcine models in biomechanics research due to cost and ease of handling considerations, and to our knowledge, head mass (for any age) and brain mass for pre-adolescent pigs have not been characterized. Additionally, there is no established relationship between head and brain mass/volume, and total body mass for the pre-adolescent pig, which may be useful due to the rapid growth that occurs during this developmental stage [7].

The CoM of the human head has been defined using both physical mechanical methods [3, 37, 39], and computed tomography (CT) imaging methods [21]. Additionally, the human head ACS is typically defined relative to the Frankfort plane which is nominally oriented horizontally [34] in the standard forward-gaze reference position, and is approximately perpendicular to the spine axis in the bipedal human [39]. The head and brain CoMs, and an associated ACS, have also been defined for adult sheep [31], but similar work has not been reported for the pig head and brain. The ACS defined by Sharkey et al. [31] using internal skull landmarks on CTs of sheep heads, corresponded to an equivalent forward-gaze head posture in this quadruped. An ACS for the pig head, with similitude to the human ACS, will improve the translational application of head kinematics data in biomechanics models using pigs. Additionally, though the MoI of cadaveric human heads have previously been characterized [3, 37], similar analysis has not been conducted for the pig.

To characterize the head and brain geometric and inertial properties of the pre-adolescent domestic pig, the aims of this study were the following: (i) to determine the relationship between head and brain mass/volume to total body mass; (ii) to determine the head and brain CoMs, and location of the AOJ relative to palpable landmarks; (iii) to define a translationally relevant ACS; and, (iv) to calculate the head and brain MoI.

## Materials and Methods

Eleven female Large White × Landrace pigs (approximately 2–4 months old) with no prior experimental protocol affecting the head, brain or cervical spine, were used. Body mass was measured prior to death on the day of imaging and ranged from 18 to 48 kg (Table 1; EZI Weigh 2, Tru-Test Datamars, TX, USA). Four pigs were imaged in vivo as part of experimental protocols for an unrelated study approved by the South Australian Health and Medical Research Institute Animal Ethics Committee (SAM22-031). These animals were anaesthetized, intubated, and mechanically ventilated during imaging. Seven cadaveric pig head and necks (dissected below the shoulders) were obtained with Animal Ethics Committee tissue request approval after the animals were humanely euthanized with sodium pentobarbital as part of separately approved unrelated procedures. The cadaveric tissue was stored at − 20 °C and imaged frozen (*N* = 5), or thawed to rectify soft tissue deformation that had occurred during storage (*N* = 2) (Table 1).

**Table 1:** Live body mass for each animal and imaging parameters for each animal (in vivo) or head/neck specimen (frozen/thawed)

Subject	Live body mass [kg]	State during imaging	Scanner placement	Voxel size [mm]
1	27	In vivo	Prone	0.47 × 0.47 × 1.00
2	29	In vivo	Prone	0.41 × 0.41 × 1.00
3	22	In vivo	Prone	0.49 × 0.49 × 0.70
4	18	In vivo	Prone	0.39 × 0.39 × 1.00
5	44	Frozen	Supine	0.53 × 0.53 × 0.60
6	31	Thawed	Supine	0.52 × 0.52 × 0.60
7	30	Thawed	Supine	0.54 × 0.54 × 0.60
8	48	Frozen	Supine	0.58 × 0.58 × 0.60
9	36	Frozen	Supine	0.55 × 0.55 × 0.60
10	39	Frozen	Supine	0.59 × 0.59 × 0.60
11	41	Frozen	Supine	0.59 × 0.59 × 0.60
Mean	33			
SD	9			

### CT Imaging and Segmentation

Computed tomography (CT) scans of the head and upper neck were obtained with the in vivo animals positioned prone and the cadaveric specimens positioned supine using a Biograph mCT scanner (Siemens Healthineers AG, Elangen, Germany). All subjects were scanned with a closed mouth where the upper and lower dentition approximated one another.

Scans were reconstructed with an in-plane resolution and slice thickness ranging from 0.39 to 0.59 mm and 0.6 to 1.0 mm, respectively (Table 1). In-plane resolution varied based on the axial field of view selected for the 512 × 512 matrix.

**Fig. 1:**
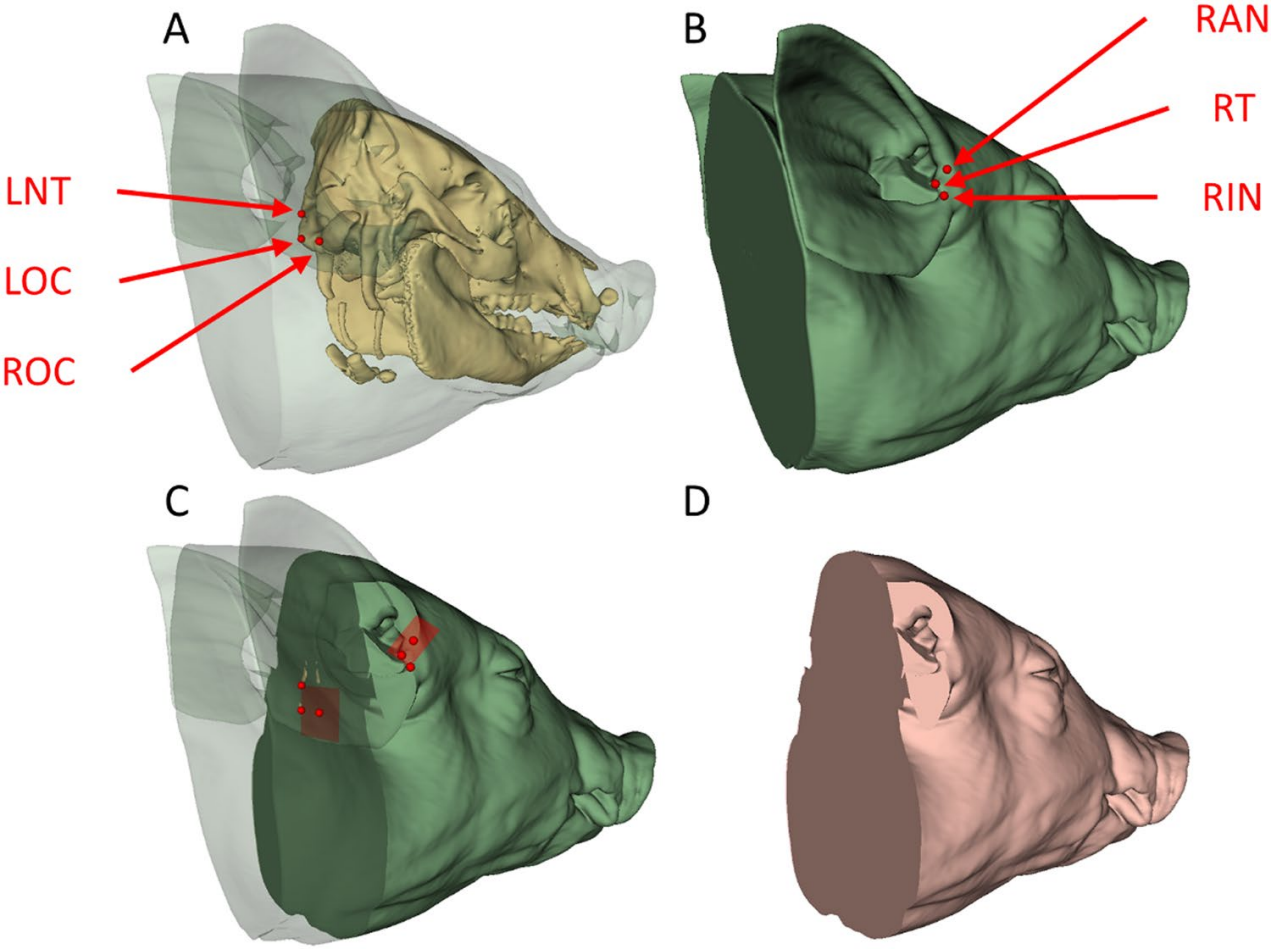
**A** Head-neck cutting plane defined by the left nuchal tubercle (LNT), left occipital condyle (LOC), and right occipital condyle (ROC). **B** Right ear cutting plane defined by the right anterior notch of auricle (RAN), right tragus (RT), and right intertragal notch (RIN). Left ear cutting plane defined similarly, not shown. **C** Head-neck and ear cutting planes applied to head mask. **D** Final head model

Open source image analysis software (3D Slicer 5.2.1, slicer.org) [12] was used to segment the head and brain from the CT scans. Initial head masks, which included soft tissue, brain, and bone, were created by segmenting the CT images at a global threshold of − 300 Hounsfield units (HU). The head masks for the in vivo subjects were further segmented via thresholding to remove the endotracheal tubes. To segment the brain, a global threshold of 250 HU was used to create a bone mask enveloping the brain, then voxels with HU less than 250 HU within this volume were defined as brain tissue. All automatic segmentations were corrected manually where necessary to remove erroneously segmented voxels (e.g., towels and blankets under the animal). To delineate the border of the head and brain at the neck, a plane was defined on the bone mask, using the most caudal aspect of the right and left occipital condyles and the nuchal tubercles (Fig. 1A). Ear cutting planes were also defined bilaterally on the head masks by the tragus, intertragal notch (incisura intertragica), and anterior notch of auricle (incisura anterior) (Fig. 1B). First the head-neck cutting plane was applied to the head and brain masks, then the ear cutting planes were applied to the head mask (Fig. 1C) to obtain the final segmented head (Fig. 1D). The ear cutting planes were applied to the head masks as the positioning of the ears, with respect to symmetrical intra-subject and consistent inter-subject placement, was not considered during imaging. Using the final head and brain models, the three-dimensional position (in CT coordinates) and HU of all voxels were exported from 3D Slicer for further analysis.

### Mass, Center of Mass, and Atlanto-Occipital Joint

Using a custom MATLAB program (R2019b, Mathworks, Natick, MA, USA), the mass of each voxel was calculated using its HU, volume, and a HU-to-density calibration equation.

To obtain the CT scanner and scan parameter-specific calibration equations to convert the HU of each voxel to density (kg/m^3^), CT images were acquired of a tissue density phantom (Model 062 M, CIRS Tissue Simulation & Phantom Technology, Norfolk, VA, USA) comprising rods of known densities equivalent to air, adipose tissue, water, muscle, trabecular bone, and cortical bone. Each rod was manually segmented in each image slice, avoiding pixels at the rod border, and the mean HU was determined for each segmented rod volume (Supplementary Table S1). Calibration equations were obtained using bilinear regression, separated at the mean muscle rod HU [2, 15] (Supplementary Fig. S1). In the cadaveric specimens, some air (defined as < − 500 HU) was present in the brain. These voxels were assigned the mean density of the animal-specific brain tissue calculated without contribution from the air voxels.

Using the mass and volume of each voxel, the total mass and volume of the head and brain was calculated, respectively. Using the mass and CT coordinates of each voxel [31], the CoM of the head and brain was calculated in the CT coordinate system using Equation 1 where $\overline{x },\overline{y },\overline{z }$ are the CoM coordinates, m_i_ is the mass of each voxel, and *x*_*i*_, *y*_*i*_, *z*_*i*_ are the coordinates of each voxel. The coordinates of the AOJ was also defined by the midpoint of the most caudal aspect of the right and left occipital condyles (as defined for the head-neck cutting plane).

1 $$\begin{gathered} \overline{x} = \frac{{\mathop \sum \nolimits_{i = 1}^{n} m_{i} x_{i} }}{{\mathop \sum \nolimits_{i = 1}^{n} m_{i} }} \hfill \\ \overline{y} = \frac{{\mathop \sum \nolimits_{i = 1}^{n} m_{i} y_{i} }}{{\mathop \sum \nolimits_{i = 1}^{n} m_{i} }} \hfill \\ \overline{z} = \frac{{\mathop \sum \nolimits_{i = 1}^{n} m_{i} z_{i} }}{{\mathop \sum \nolimits_{i = 1}^{n} m_{i} }} \hfill \\ \end{gathered}$$

### Anatomical Coordinate System

**Fig. 2:**
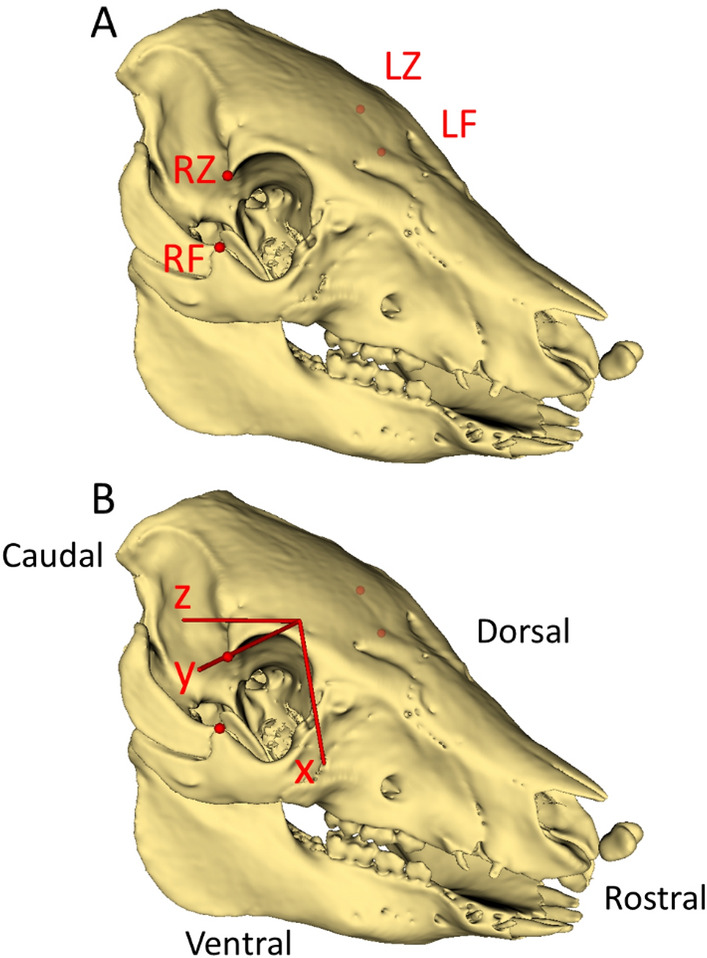
Skull of representative subject showing **A** bony landmarks used for anatomical coordinate system definition (red dots): right/left frontal process of the zygomatic bone (RF, LF) and right/left zygomatic process of the frontal bone (RZ, LZ), and **B** defined anatomical coordinate system with positive axes (red lines) and quadruped anatomical terms

To define an ACS, an ex vivo subject was palpated to identify bony landmarks that could be used to define a nominal forward-gaze plane similar to the Frankfort plane in humans. An ACS was defined using the right and left frontal process of the zygomatic bone (RF, LF) and zygomatic process of the frontal bone (RZ, LZ) [18] which were identified on the bone mask for each subject (Fig. 2A). The ACS origin was defined as the midpoint between RZ & LZ. Positive x axis was defined as pointing towards the midpoint between RF & LF, and the y axis was preliminarily defined as pointing towards RZ. An orthogonal positive z axis was defined via a cross product of the x and y axes (Fig. 2B). Finally, to ensure orthogonality of all axes, the final positive y axis was defined via a cross product of the x and z axes.

Using the CT coordinates of the bony landmarks and custom code in MATLAB, an ACS was defined for each subject, and the CoM and AOJ coordinates were subsequently transformed from CT coordinates to the ACS.

### Mass Moments of Inertia

Using a method similar to Durston et al. [11] to calculate the head and brain principal MoI, the MoI tensor (*I*_ACS_) was first calculated in the ACS (Equation 2) in MATLAB. In Equation 2, m_i_ is the mass of each voxel, and *x*_*i*,_ *y*_*i*_, *z*_*i*_ are the respective *x*, *y*, and *z* coordinates of the voxel in the ACS. Using the parallel axis theorem (Equation 3), the *I*_ACS_ tensor was then translated such that the ACS origin was coincident with the respective head or brain CoMs (*I*_CoM_). The eigenvectors and eigenvalues of the *I*_CoM_ tensor were then calculated to determine the principal axes and components of inertia, respectively.

2 $$\begin{gathered} \begin{array}{*{20}c} {I_{xx} = \mathop \sum \limits_{i = 1}^{n} m_{i} \left( {y_{i}^{2} + z_{i}^{2} } \right)}\; & {I_{xy} = - \mathop \sum \limits_{i = 1}^{n} m_{i} x_{i} y_{i} } \\ \end{array} \hfill \\ \begin{array}{*{20}c} {I_{yy} = \mathop \sum \limits_{i = 1}^{n} m_{i} \left( {x_{i}^{2} + z_{i}^{2} } \right)}\; & {I_{xz} = - \mathop \sum \limits_{i = 1}^{n} m_{i} x_{i} z_{i} } \\ \end{array} \hfill \\ \begin{array}{*{20}c} {I_{zz} = \mathop \sum \limits_{i = 1}^{n} m_{i} \left( {x_{i}^{2} + y_{i}^{2} } \right)}\; & {I_{yz} = - \mathop \sum \limits_{i = 1}^{n} m_{i} y_{i} z_{i} } \\ \end{array} \hfill \\ \end{gathered}$$

3 $$I_{{{\text{CoM}}}} = I_{{{\text{ACS}}}} - \mathop \sum \limits_{i = 1}^{n} m_{i} d^{2}$$

### Statistical Analysis

Data are reported as mean ± standard deviation (SD). For both the head and brain, linear regression was used to assess the correlation between tissue mass and body mass, and tissue volume and body mass (*α* = 0.05). Statistical analyses were performed in SPSS Statistics (v28, IBM, Illinois, USA).

## Results

The head and brain had mean masses of 2533 ± 646 g and 103 ± 9 g, and mean volumes of 2413 ± 635 cm^3^ and 100 ± 10 cm^3^, respectively. On average, the head and brain constituted 7.80 ± 0.79% and 0.33 ± 0.08% of the body mass, respectively.

**Fig. 3:**
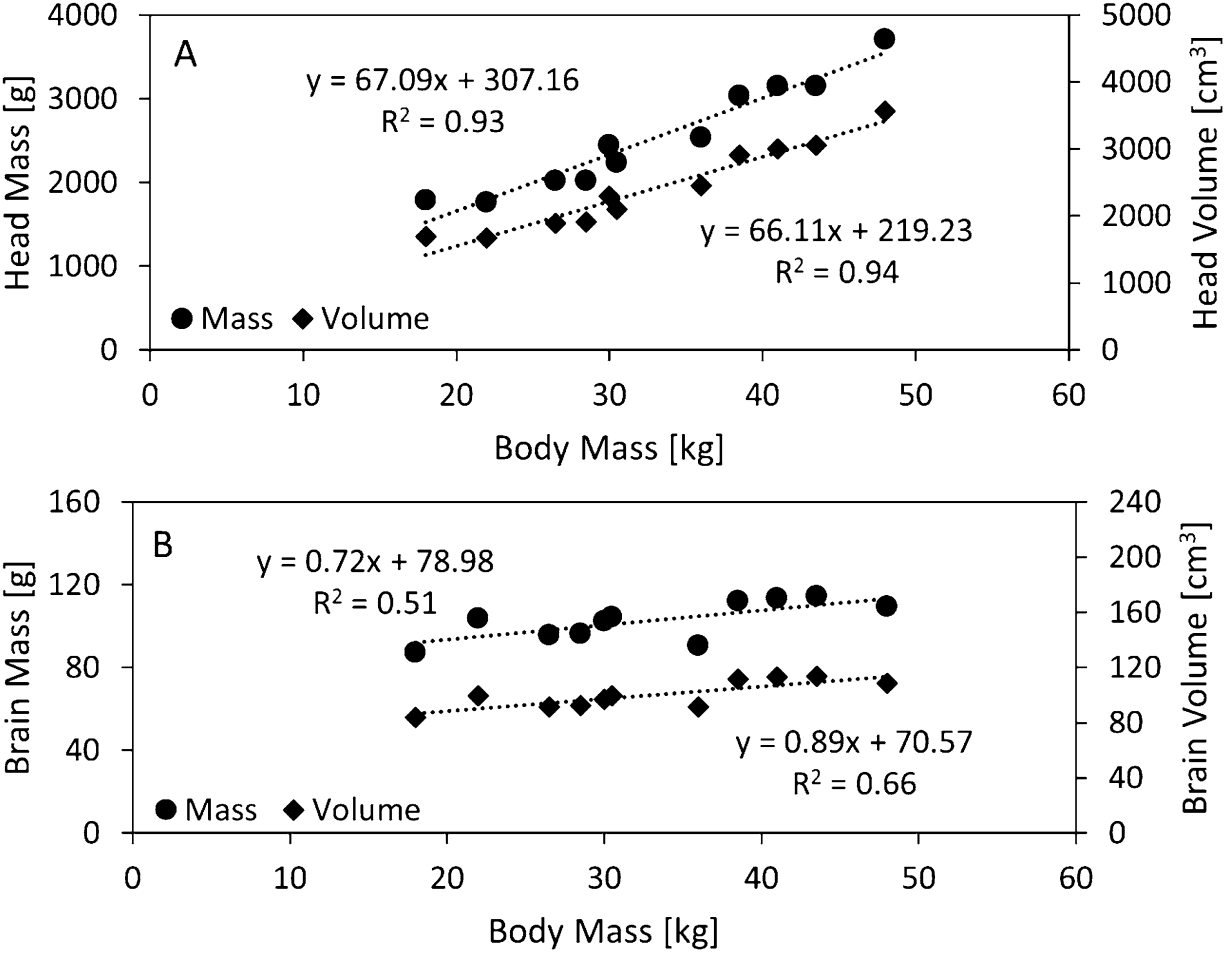
Linear regression of the head (**A**)/brain (**B**) mass and body mass, and head (**A**)/brain (**B**) volume and body mass

Head mass and head volume were linearly correlated with body mass (*p* < 0.001), and body mass explained 93% and 94% of the variation in the head mass and volume, respectively (Fig. 3A). Similarly, brain mass and brain volume were linearly correlated with body mass (*p* = 0.013 and 0.002, respectively), and body mass explained 51% and 66% of the variation in the brain mass and volume, respectively (Fig. 3B). Tabulated data from Fig. 3, and the associated regression model outputs are in Supplementary Table S2 and S3, respectively.

**Fig. 4:**
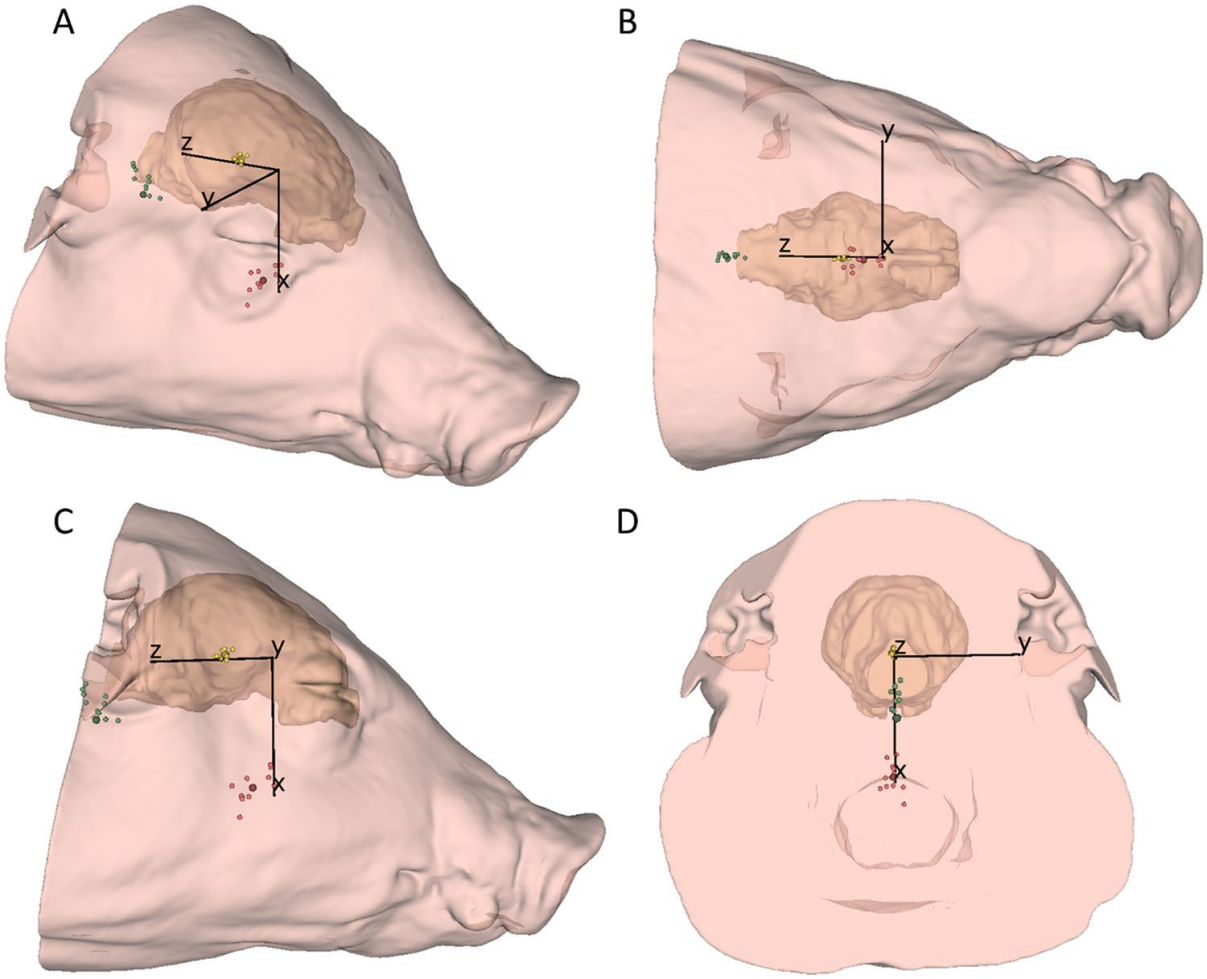
Center of mass of the head (small red spheres), center of mass of the brain (small yellow spheres), and atlanto-occipital joint (small green spheres) of each animal in the anatomical coordinate system of a representative animal from **A** oblique, **B** below/*y*–*z* plane, **C** right/*x*–*z* plane, and **D** rear/*x*–*y* plane view. Mean head center of mass, brain center of mass, and atlanto-occipital joint locations are indicated by the larger/darker red, yellow, and green spheres, respectively. A video of the 3D model in this figure can be found in Supplementary Video S1

The head CoMs for all subjects were primarily ventral to the ACS origin and the mean CoM was positioned 47.3 ± 6.0 mm away (Table 2; Fig. 4). The mean head principal MoI about the head CoM were 61.74 ± 25.26, 96.09 ± 41.60, and 109.73 ± 50.18 kg cm^2^, respectively (Table 2).

**Table 2:** Head center of mass (CoM) coordinates (in the anatomical coordinate system, ACS) and principal moments of inertia (MoI; origin at head CoM), for each animal

Subject	CoM coordinates in ACS [mm]			CoM distance to ACS origin [mm]	Principal MoI_CoM_ [kg cm^2^]		
	*x*	*y*	*z*		*I*_1_	*I*_2_	*I*_3_
1	49.3	− 0.4	9.3	50.2	38.55	69.53	72.03
2	45.5	− 0.5	0.3	45.5	46.81	62.85	71.61
3	42.6	− 0.5	10.0	43.8	32.84	50.84	54.00
4	42.7	− 1.0	2.3	42.8	40.60	48.30	54.08
5	50.5	− 5.7	11.8	52.2	83.11	139.00	161.16
6	39.2	− 3.0	1.0	39.4	55.98	71.90	86.87
7	38.1	0.9	1.3	38.1	66.66	79.21	92.85
8	56.3	3.3	12.8	57.8	117.66	172.34	210.23
9	45.7	− 3.7	15.4	48.3	45.03	99.44	114.32
10	49.6	− 3.2	12.3	51.2	73.53	125.58	139.91
11	49.9	3.4	11.9	51.4	78.39	137.99	149.99
Mean	46.3	− 0.9	8.0	47.3	61.74	96.09	109.73
SD	5.4	2.8	5.6	6.0	25.26	41.60	50.18

The brain CoMs for all subjects were primarily caudal to the ACS origin and the mean CoM was positioned 17.1 ± 1.7 mm away (Table 3; Fig. 4). The mean brain principal MoI about the brain CoM were 0.24 ± 0.04, 0.55 ± 0.09, and 0.57 ± 0.10 kg cm^2^, respectively (Table 3). The head and brain inertia tensor (in the ACS with origin at the respective head or brain CoM) can be found in Supplementary Table S4 and S5, respectively. The unit vectors for the principal axes for the head and brain can also be found in Supplementary Table S6 and S7, respectively.

**Table 3:** Brain center of mass (CoM) coordinates (in the anatomical coordinate system, ACS) and principal moments of inertia (MoI; origin at brain CoM), for each animal

Subject	CoM coordinates in ACS [mm]			CoM distance to ACS origin [mm]	Principal MoI_CoM_ [kg cm^2^]		
	*x*	*y*	*z*		*I*_1_	*I*_2_	*I*_3_
1	− 1.1	− 0.1	16.0	16.0	0.23	0.46	0.47
2	0.0	0.0	18.2	18.2	0.23	0.46	0.47
3	− 3.4	− 0.3	13.9	14.3	0.21	0.54	0.58
4	0.3	0.4	16.1	16.1	0.20	0.40	0.41
5	− 2.9	− 0.1	17.6	17.8	0.27	0.65	0.69
6	− 0.7	− 0.3	15.8	15.9	0.26	0.52	0.53
7	− 1.8	− 1.3	17.0	17.1	0.23	0.54	0.55
8	− 1.4	− 0.4	19.3	19.3	0.26	0.62	0.63
9	− 3.6	0.0	16.7	17.1	0.14	0.48	0.53
10	− 1.4	− 0.8	20.0	20.1	0.28	0.65	0.66
11	− 2.2	− 0.7	16.3	16.5	0.28	0.68	0.70
Mean	− 1.7	− 0.3	17.0	17.1	0.24	0.55	0.57
SD	1.3	0.5	1.7	1.7	0.04	0.09	0.10

The AOJ locations were ventrocaudal to the ACS origin and the mean AOJ was positioned 65.1 ± 3.3 mm away (Table 4; Fig. 4).

**Table 4:** Atlanto-occipital joint (AOJ) coordinates (in anatomical coordinate system, ACS), for each animal

Subject	AOJ coordinates in ACS [mm]			AOJ distance to ACS origin [mm]
	*x*	*y*	*z*	
1	14.7	0.7	60.4	62.1
2	20.4	1.3	60.1	63.4
3	11.9	1.6	59.5	60.7
4	20.8	0.1	55.8	59.5
5	14.1	1.7	66.8	68.3
6	18.2	− 0.6	62.3	64.9
7	17.1	− 0.8	62.7	65.0
8	11.3	− 1.2	67.5	68.5
9	7.3	2.3	66.3	66.8
10	9.8	0.3	67.7	68.4
11	9.4	− 0.1	67.8	68.4
Mean	14.1	0.5	63.4	65.1
SD	4.6	1.1	4.1	3.3

## Discussion

Pigs are a common animal model in several injury biomechanics applications. To aid comparisons and translation between porcine models and human biomechanical surrogates and human subjects, the geometric and inertial properties of the domestic pig head and brain in an ACS were characterized in this study.

**Fig. 5:**
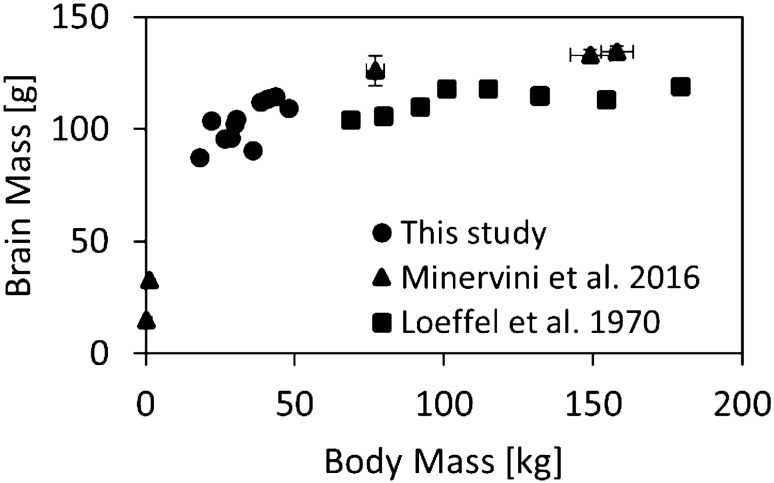
Brain mass vs body mass for domestic pigs between neonate and adult development stages from literature (mix of Landrace, Large White and their cross-breed, triangles [24]; and, mix of Duroc, Berkshire, and Chester-White breeds, squares [20]) including data from this study (individual animals, circles). Previously published data refers to mean data for fresh ex vivo tissue measured physically; data are mean ± standard error of mean where available

The mass and volume of the head and brain were positively linearly correlated with body mass in these animals weighing 18–48 kg. On average, the pig head and brain constituted 7.80% and 0.33% of the body mass, respectively. The relationships in Fig. 3 can be used to estimate the mass and volume of the pig head and brain using body mass for pre-adolescent pigs of similar mass. The brain masses reported herein address the gap in literature for pre-adolescent domestic pigs (Fig. 5). Previous studies of adolescent domestic pigs have reported mean brain masses of 104 g (68.7 kg mean body mass; mix of Duroc, Berkshire, and Chester-White breeds) [20] and 126 g (77 kg mean body mass; Large White) [24], which are larger than the 103 g mean brain mass for the smaller pigs (33 kg mean body mass) in this study, but correspond to a lower ratio (0.15, 0.16%, respectively, compared to our value of 0.33%) of total body mass. The head-to-body-mass ratios for the pigs in this study are similar to adult humans; for human cadavers (38 male, 2 female, mean age 65.2 years) this ratio is approximately 6.6% [39]. However, compared to the average adult human brain-to-body-mass ratio (2.3% [23]), the pre-adolescent pig brain is considerably smaller.

The ACS defined for the pig head uses four externally palpable landmarks, which allows for non-invasive ACS definition in in vivo porcine models (for example with a coordinate measuring machine or motion capture system) without the need for 3D medical imaging. The standard coordinate system for human head kinematics (particularly in automotive safety research) defines positive x, y and z axes as pointing in the anterior, right lateral, and inferior directions relative to the head, respectively [40]. In this human-centric coordinate system, the x-y axes define the Frankfort plane which is nominally horizontal in a forward-facing gaze [34] and perpendicular to the spine axis. The pig ACS was defined with positive *x*, *y*, and *z* axes pointing in the ventral, right lateral, and caudal directions relative to the head, respectively. By this definition, the “Frankfort plane” in the pig corresponds to the *y*–*z* plane and is approximately horizontal in the prone/supine position in a forward-facing gaze, while the plane nominally perpendicular to the spine axis constitutes the* x*–*y* plane as in humans. This ACS definition aims to produce a translatable coordinate system between pigs and humans by considering the orientation of the spine axis in the quadrupedal pig.

The head and brain CoMs were primarily ventral and caudal to the ACS origin, respectively, with the brain CoM located closer to the ACS origin. The brain CoMs were clustered closer to the mean, with a distance of 1.9 ± 0.8 mm from the mean, while the head CoMs were located 7.5 ± 2.6 mm from the mean. The larger scatter in the head CoM location likely reflects greater variability in the amount and distribution of soft tissue external to the skull, related to scanning position, total body weight, and animal-specific anthropometry. Similar to the findings in sheep [31], the head and brain CoMs in the pig were not coincident and were separated by 49.3 ± 4.8 mm. Given the distance between the pig head and brain CoMs, reporting kinematics at their respective locations may be prudent rather than assuming an interchangeable or coincident CoM as previously applied in non-human primate TBI research [1].

The AOJs were primarily ventrocaudal to the ACS origin and were located 5.6 ± 2.1 mm from the mean AOJ location. The AOJ was located 49.2 ± 2.4 mm and 64.7 ± 4.1 mm from the brain and head CoMs, respectively. The distance between the AOJ and head CoM in the pig was greater than in humans (53.3 ± 10.8 mm [39]). The distance between the head/brain CoM and AOJ in the pig again points to the importance of reporting and comparing kinematics at their respective locations.

The mean head and brain principal MoI ranged from 61.74 to 109.73 kg cm^2^, and 0.24 to 0.57 kg cm^2^, respectively. Compared to the cadaveric adult human head, the mean head principal MoI for the pigs in this study are smaller; the mean human head principal MoI has been reported to range from 164.0 to 200.8 kg cm^2^ (6 male; mean age 54.3 years; mean head mass 3.99 kg) [5] and from 148.4 to 223.4 kg cm^2^ (19 male, 2 female; mean age 42.2 years; mean head mass 4.3 kg) [3]. The smaller principal MoI for the pig head can be explained by the smaller head mass (mean 2.5 kg) as head mass and MoI are linearly correlated [39]. Based on these findings, for head MoI comparable to humans, larger pigs than used in this study may be more translationally relevant for studies concerned with rotational kinematics.

The data reported herein can be applied in porcine injury models where head or brain kinematics are measured using either a subject-specific or a generic approach, as described by Sharkey et al. [31]. For the former, animal-specific CT images can be used to identify the bony landmarks described here to define the animal-specific ACS and to calculate the head or brain CoMs and AOJ locations for transformation of kinematics data. For the generic approach, the animal’s ACS can be defined by recording the 3D locations of the palpable bony landmarks in the experiment coordinate system (using a coordinate measuring machine or motion capture system) and the head and brain CoM and AOJ locations can be estimated from the mean values reported in Tables 2, 3, and 4. Although the animal-specific method more accurately defines the head or brain CoMs and AOJs, this analysis is more time and labor intensive and the generic approach may be sufficiently accurate in some applications and necessary where CT or other appropriate imaging is not available or practicable. Using standard head CoM or mass properties based on population-based anthropometric data is common in human volunteer and post-mortem subject testing of head kinematics where obtaining subject-specific properties is not practicable [9, 38].

This study has several limitations. The semi-automated segmentation and selection of landmarks for defining cutting planes and the ACS involved subjectivity. However, all image processing was conducted by one operator (author NS) to eliminate inter-operator variability. The head CoM analysis excluded the ears because they were inconsistently positioned during imaging. However, the effect of the ears on the head CoM position is likely minimal as the ears have low mass (the portion of both ears segmented by the ear cutting planes were 0.90 ± 0.62% of total head mass), are symmetric about the sagittal midline, and are typically taped to the head during head injury testing [31]. Additionally, the consistent positioning of the tongue was not considered (can vary due to intubation).

The head and brain CoM coordinates were reported in an ACS defined by landmarks placed on the bone mask. In contrast, to use the generic CoM locations in in vivo porcine models during an experiment, landmarks will need to be identified via external palpation through skin and subcutaneous soft tissue which can affect the accuracy of landmark digitization (although an invasive approach could expose the landmarks). However, since the ACS origin lies at the midpoint of bilateral landmarks, bias from soft tissue over the bony landmarks should be approximately equal bilaterally, assuming mid-sagittal symmetry.

Fluid loss, morphological changes, and freezing/thawing of the ex vivo subjects could have affected the head and brain densities and subsequently the mass, CoM and MoI calculations [4, 16]. Qualitatively, the CT images of the ex vivo subjects were similar to those of the animals imaged in vivo. Though some air was present in the brains of the ex vivo subjects, these voxels constituted a small volume (0.39 ± 0.48% of total brain volume) and were treated by being assigned the average density of the brain (excluding air voxels). Overall, using two-sample *t* tests, the head and brain CoM components (except for the brain CoM y component) did not differ between the in vivo and ex vivo subjects (head *x*, *y*, and *z* *p* values: 0.51, 0.71, 0.26; brain *x*, *y*, and *z* *p* values: 0.37, 0.05, 0.18). Similar comparisons of head/brain mass and MoI between in vivo and ex vivo subjects were not conducted as the in vivo and ex vivo groups in this study had disparate total body mass (mean 24 vs. 38 kg, *t* test *p* value: 0.003) which would confound the comparisons.

This study exclusively used a CT imaging approach to calculate head and brain mass, CoM locations and MoI. Although physical techniques have conventionally been used to determine the human head CoM and MoI [3, 37, 39], CT methods similar to those used herein have previously been validated against the physical methods [11, 29]. Furthermore, determining the brain CoM through physical methods requires isolating the brain from the head, which would preclude defining its CoM relative to a head ACS.

Finally, this study used female pigs of one domestic strain with body mass ranging from 18 to 48 kg. Domestic pigs (male or female) of this size are typically between 8 and 13 weeks old [6, 7] and are at a post-weaning/pre-adolescent stage (female pigs reach puberty at approximately 25 weeks) [7, 28]. Since the reported results could be sex, breed, and size dependent, these factors should be considered in the application of these findings. However, our dataset includes the most common sex and size of pig used for whiplash injury and TBI models, and previous studies have used pigs from neonatal to adolescent developmental stages [8, 36].

In summary, this study reports head and brain geometric and inertial properties, including center of mass and mass moment of inertia, for the pre-adolescent domestic pig. A head anatomical coordinate system with a pig-equivalent Frankfort plane is also defined using externally palpable bony landmarks. Application of this study to report head and brain kinematics and kinetics in an established anatomical coordinate system can aid the comparison between porcine studies, and the translation of data from porcine models to other animal models, human biomechanical models, and human subjects.

### Supplementary Information

Below is the link to the electronic supplementary material.

Supplementary file1 (PDF 424 kb)

Supplementary file2 (MP4 1591 kb)

## Data Availability

The data that support the findings of this study are publicly available in the Figshare repository, as part of this record: https://doi.org/10.25909/c.6686636 [35].
