# Supplementary material for: Geometric and Inertial Properties of the Pig Head and Brain in an Anatomical Coordinate System
Source: Ann Biomed Eng. 2023 Jun 26;51(11):2544–53. doi: 10.1007/s10439-023-03294-y (PMC10598157; doi:10.1007/s10439-023-03294-y)
Supplement: Supplementary file 1 — Supplementary file1 (PDF 424 kb) [file 10439_2023_3294_MOESM1_ESM.pdf]

SUPPLEMENTARY MATERIAL

Table S1: Known density and determined Hounsfield units (HU) of the tissue density phantom rods for the *in vivo* and *ex vivo* scans obtained at 100 and 120 kV, respectively.

		In Vivo Scans (100 kV)	Ex Vivo Scans (120 kV)
**Phantom Rod**	**Density [kg/m^3]**	**Average HU**	**Average HU**
Lung (inhale)	205	-810.99	-809.37
Lung (exhale)	507	-510.92	-513.09
Adipose	960	-64.93	-58.83
Water	1000	0.93	-0.62
Muscle	1060	53.14	51.73
Trabecular bone	1160	296.47	260.60
Dense Bone	1530	1089.4	951.48

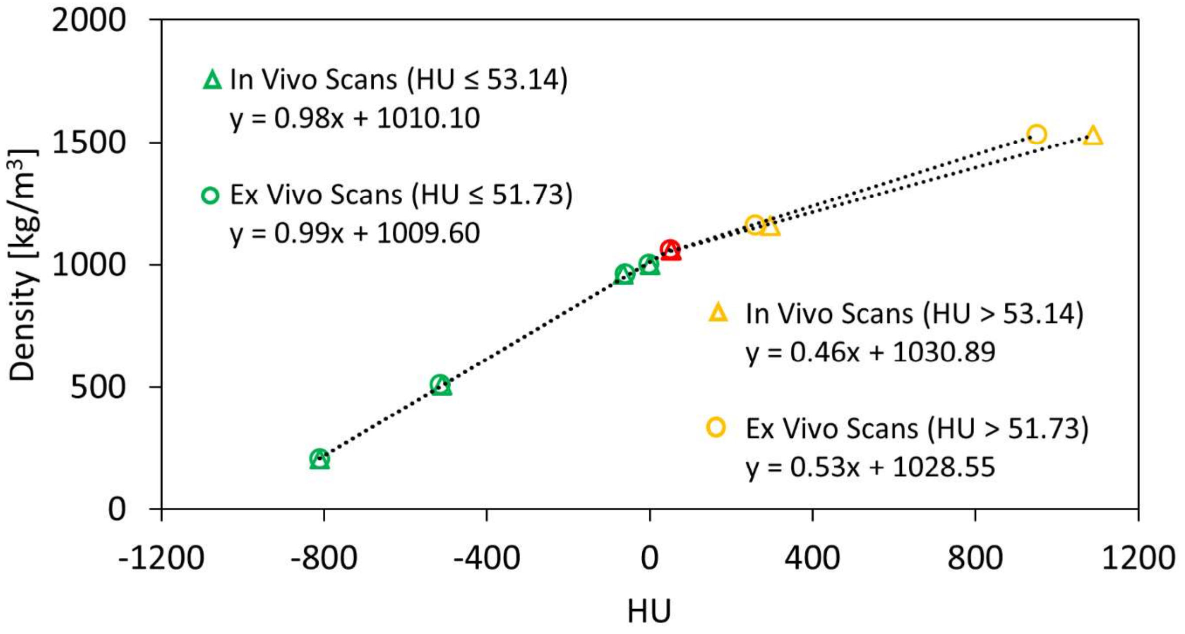

Figure S1: Soft (green) and hard (yellow) tissue phantom calibration equations for the *in vivo* (triangle) and *ex vivo* (circle) scans using density and Hounsfield units (HUs) from Table S1. The muscle HUs (red triangle and circle) were used to separate the respective *in vivo* and *ex vivo* bilinear regressions.

Table S2: Body mass, head mass, head volume, brain mass, and brain volume for each subject. SD = standard deviation.

Subject	Body Mass [kg]	Head Mass [g]	Head Volume [cm^3]	Brain Mass [g]	Brain Volume [cm^3]
1	27	2024	1894	96	91
2	29	2020	1916	96	93
3	22	1766	1667	104	99
4	18	1784	1689	87	84
5	44	3151	3054	114	114
6	31	2239	2099	104	100
7	30	2446	2297	102	97
8	48	3710	3560	109	109
9	36	2536	2455	91	91
10	39	3037	2905	112	111
11	41	3153	3006	114	113
**Mean**	33	2533	2413	103	100
**SD**	9	646	635	9	10

Table S3: Intercept, slope, 95% confidence intervals, standard errors, and p-values for the head/brain mass vs body mass and volume vs body mass linear regressions.

			95% Confidence Interval			
		**Value**	**Lower Bound**	**Upper Bound**	**Standard Error**	**p-value**
**Head Mass vs Body Mass**	**Intercept**	307.16	-154.96	769.28	204.28	0.167
	**Slope**	67.09	53.64	80.55	5.95	< 0.001
**Head Volume vs Body Mass**	**Intercept**	219.23	-219.51	657.96	193.95	0.288
	**Slope**	66.11	53.33	78.88	5.65	< 0.001
**Brain Mass vs Body Mass**	**Intercept**	78.98	60.96	97.01	7.97	< 0.001
	**Slope**	0.72	0.19	1.24	0.23	0.013
**Brain Volume vs Body Mass**	**Intercept**	70.57	53.88	87.27	7.38	< 0.001
	**Slope**	0.89	0.41	1.38	0.21	0.002

Table S4: Head mass moment of inertia tensor in the anatomical coordinate system with origin at the head center of mass.

	Moment of Inertia Tensor (I_{CoM}) [kg·cm^2]					
**Subject**	I_{xx}	I_{yy}	I_{zz}	I_{xy}	I_{xz}	I_{yz}
1	61.117	69.742	49.247	1.296	-15.489	0.541
2	62.275	70.754	48.235	2.631	4.673	0.228
3	45.195	50.852	41.633	1.047	-10.357	0.511
4	48.920	53.333	40.726	1.956	0.909	-0.367
5	116.965	131.845	134.459	21.749	-31.715	7.052
6	76.935	74.172	63.637	6.295	12.220	0.713
7	87.795	79.789	71.132	-2.364	9.711	-1.496
8	157.341	166.024	176.867	-14.373	-41.981	-10.071
9	80.746	97.405	80.639	13.244	-31.949	6.410
10	110.335	123.673	105.007	13.282	-30.070	6.844
11	125.429	135.549	105.386	-13.299	-30.872	-10.245
**Mean**	88.459	95.740	83.361	2.860	-14.993	0.011
**SD**	35.304	38.005	43.371	10.849	19.460	5.895

Table S5: Brain mass moment of inertia tensor in the anatomical coordinate system with origin at the brain center of mass.

	Moment of Inertia Tensor (I_{CoM}) [kg·cm^2]					
**Subject**	I_{xx}	I_{yy}	I_{zz}	I_{xy}	I_{xz}	I_{yz}
1	0.466	0.465	0.235	0.000	0.039	0.006
2	0.459	0.466	0.234	-0.001	-0.018	-0.010
3	0.521	0.540	0.266	-0.002	0.137	0.003
4	0.395	0.406	0.202	0.000	-0.022	-0.010
5	0.641	0.646	0.317	0.001	0.132	0.001
6	0.519	0.521	0.261	0.000	0.037	0.012
7	0.533	0.534	0.253	-0.005	0.063	0.049
8	0.599	0.621	0.296	0.001	0.103	0.016
9	0.465	0.483	0.214	0.001	0.149	-0.011
10	0.636	0.657	0.306	-0.001	0.087	0.023
11	0.673	0.681	0.308	-0.004	0.105	0.024
**Mean**	0.537	0.547	0.263	-0.001	0.074	0.009
**SD**	0.090	0.091	0.040	0.002	0.059	0.018

Table S6: Unit vectors for principal axes of inertia of the head (in the anatomical coordinate system with origin at the head center of mass).

	$\overline{v}_1$			$\overline{v}_2$			$\overline{v}_3$		
**Subject**	**x**	**y**	**z**	**x**	**y**	**z**	**x**	**y**	**z**
1	0.567	-0.038	0.823	0.244	-0.946	-0.212	0.787	0.321	-0.527
2	-0.293	0.023	0.956	0.907	-0.310	0.285	0.303	0.950	0.070
3	0.644	-0.059	0.763	0.078	-0.987	-0.142	0.761	0.151	-0.631
4	-0.119	0.047	0.992	-0.927	0.351	-0.128	-0.355	-0.935	0.002
5	-0.750	0.410	-0.519	-0.118	-0.855	-0.505	-0.651	-0.317	0.690
6	-0.531	0.151	0.834	0.285	-0.895	0.344	0.798	0.420	0.433
7	-0.415	0.029	0.910	-0.201	-0.978	-0.060	0.888	-0.208	0.411
8	0.739	0.341	0.582	0.250	-0.940	0.232	-0.626	0.026	0.780
9	0.696	-0.258	0.671	-0.060	0.909	0.412	-0.716	-0.327	0.617
10	0.666	-0.271	0.695	-0.102	0.890	0.445	-0.739	-0.367	0.565
11	0.581	0.273	0.767	0.188	0.872	-0.452	-0.792	0.407	0.455

Table S7: Unit vectors for principal axes of inertia of the brain (in the anatomical coordinate system with origin at the brain center of mass).

	$\overline{v}_1$			$\overline{v}_2$			$\overline{v}_3$		
**Subject**	**x**	**y**	**z**	**x**	**y**	**z**	**x**	**y**	**z**
1	-0.161	-0.023	0.987	-0.158	0.987	-0.003	-0.974	-0.157	-0.163
2	0.079	0.043	0.996	0.994	0.067	-0.081	-0.070	0.997	-0.038
3	-0.399	-0.012	0.917	0.010	1.000	0.017	-0.917	0.016	-0.399
4	0.111	0.048	0.993	0.990	-0.096	-0.106	0.091	0.994	-0.058
5	-0.335	-0.003	0.942	-0.024	1.000	-0.005	-0.942	-0.024	-0.335
6	-0.138	-0.045	0.989	-0.406	0.914	-0.015	-0.903	-0.404	-0.144
7	-0.206	-0.160	0.965	-0.545	0.838	0.023	-0.813	-0.521	-0.260
8	-0.292	-0.041	0.956	-0.427	0.900	-0.091	-0.856	-0.435	-0.280
9	-0.421	0.031	0.907	-0.075	-0.997	-0.001	0.904	-0.068	0.422
10	-0.238	-0.060	0.969	-0.735	0.664	-0.139	-0.635	-0.746	-0.203
11	-0.258	-0.060	0.964	-0.119	0.992	0.030	-0.959	-0.107	-0.263
